# Oxidative DNA damage induced by ROS-modulating agents with the ability to target DNA: A comparison of the biological characteristics of citrus pectin and apple pectin

**DOI:** 10.1038/s41598-018-32308-2

**Published:** 2018-09-17

**Authors:** Fahimeh Salehi, Hossein Behboudi, Gholamreza Kavoosi, Sussan K. Ardestani

**Affiliations:** 1 Institute of Biochemistry and Biophysics, Department of Biochemistry, University of Tehran, Tehran, Iran; 2 Institute of Biotechnology, Shiraz University, Shiraz, Iran

## Abstract

DNA targeting anticancer agents have been very successful in clinic, especially, when used in combinatorial therapy. But unfortunately, they often exhibit high levels of toxicity towards normal cells. Hence, much effort has been put into finding agents with more selectivity, and less toxicity. Pectins are natural polysaccharides, and beneficial nutritional fibers that have attracted attentions due to their antitumor properties. However, their molecular targets, and mechanism of action are widely unknown. Here, we have reported that citrus pectin (CP) and apple pectin (AP) selectively suppress viability in MDA-MB-231, MCF-7 and T47D human Breast cancer cells, while non-toxic to L929 normal cells. Upon CP, and AP treatments, cancer cells’ ROS content increased rapidly, and led to the collapse of the mitochondrial transmembrane potential which functions upstream of the caspase-dependent apoptosis. CP and AP treated cancer cells were also arrested at the S and G1 or G2/M phases of the cell cycle, respectively. Furthermore, mRNA expression of Galectin-3 (a multi-functional lectin involved in cell adhesion, cell cycle, and apoptosis) reduced in both CP and AP treated cells. Growth inhibition of MDA-MB-231 cells by CP, and AP was concomitant with DNA damage (oxidation, and strand breaks). In this context, in an effort to clarify the mechanism of action, we showed that CP, and AP are able to interact with DNA. The strength and mode of DNA binding were established by spectroscopy techniques. We demonstrated that CP, and AP bind to dsDNA by intercalation, and groove binding/partial intercalation, respectively. In conclusion, our findings suggest that CP, and AP induce apoptosis in MDA-MB-231 cells by increasing the release of ROS, which may be related to the mitochondrial apoptosis pathway, and direct interactions with DNA. Our data indicate that these compounds may be potentially useful in cancer treatment.

## Introduction

According to the World Health Organization (WHO), Breast cancer with about half a million death, and nearly 1.7 million new cases annually accounts for 25.2% of cancer cases, and is the most common malignancy among women. Almost 15% of Breast cancer patients die after diagnosis, which ranks it in the second place in mortality after lung cancer^1,2^. Despite advances in earlier diagnosis, and improvements in specific treatments, Breast cancer mortality has declined no more than 30% during the past twenty years. One reason for this is the development of drug resistance in cancer cells treated with single targeted drugs, which creates a feedback regulation in the cancer cells. Triple-negative Breast cancer cells (lacking estrogen, progesterone and Her2/neu receptors) outstanding resistance to common therapies, poor prognosis, and fast proliferation are  the other reasons^3,4^. Therefore, finding a way to hinder the fast proliferating Breast cancer cells by targeting multiple intracellular signaling pathways seems an effectual therapeutic approach towards curing this disease^5,6^.

Since the signaling pathways require unimpaired access to genetic codes to enact their action, Integrity of genomic DNA is critical for the appropriate function, and proliferation of the cells. Accumulation of unrepaired DNA damages is sensed at cell cycle checkpoints, and activates a series of proteins, which induce cell cycle arrest to block the transfer of damaged DNA to daughter cells during mitosis. In cancer cells, DNA repair processes are not as efficient as in normal cells, and more importantly, cell cycle check points are ignored, which allows cancer cells to proliferate at high speeds. However, fast proliferation makes cancer cells more susceptible to DNA damages, because, the replication of highly damaged DNA may increase the probability of cell death^7,8^. Hence, targeting cancer cells’ genomic DNA with minimum collateral damage to normal cells seems very applicable to stop cancer progression. One approach to achieve this goal is the recognition, and characterization of small molecules with the ability to interact with DNA, which may yield valuable information for the design, and development of new therapeutic agents, and provide an appropriate rational platform for designing new DNA targeting drugs^9–11^.

Recently, there has also been an interest in characterization of agents with the ability of increasing intracellular ROS production as an efficient way to eliminate cancer cells^12,13^. Excessive amounts of ROS within the cell cause oxidative stress, that besides profound destructive effects on cellular ingredients inflict its influence on mitochondria by jeopardizing its membrane integrity, membrane potential, and respiratory chain^14–16^. Moreover, ROS overproduction disturb the delicate balance between the members of Bcl2 family proteins, which act as anti- and proapoptotic factors, resulting in mitochondrial membrane portion, and release of cytochrome C, and other apoptogenic factors^17^. The ultimate consequence of all of these events is induction of apoptosis in a caspase dependent manner^18^. Currently, multiple chemotherapeutic drugs are known that cause DNA damage, and subsequent induction of apoptosis.

Carbohydrates are abundant in plants, and serve not only as a source of energy but also have roles in regulating biological processes, and contain numerous compounds with pharmaceutical values. There are pieces of evidence that polysaccharides from fruits have the ability to alter the signaling pathways in cancer cells. Pectins are complex branched polysaccharides rich in galactoside residues which are extracted commercially from pulp waste during fruit juice production (e.g. from apple pomace, and citrus peel)^19,20^. Recent studies indicate that plant pectins are effective against various cancers^21^. Pectins are capable of inducing apoptosis in cancer cells without having any adverse effect on normal cells, which make them good candidates in drug research. Previous studies, both *in vitro* and *in vivo*, demonstrated that modified pectins by high pH, and temperature possess chemo preventive properties against cancer cells in Breast, colon, prostate, melanoma, and multiple myeloma cancers. Apple contains a relatively modest amount of pectin, and pectic acid is the main pectin component in it. Apple fruit whole extract has been shown to be able to prevent mammary cancer in rat models^22^. Although, Apple Pectin (AP) or Citrus Pectin (CP) cannot completely prevent metastatic tumor formation, but, recent findings indicate that CP, AP, and pectic polymer are able to induce apoptosis in various cancer cells via inhibiting MAP kinase activation, and NF-kB inactivation^23^.

Multiple biomarkers have been introduced for Breast cancer. One of them is Galectin-3 (Gal-3), an endogenous lectin molecule and a member of the galectin family, that have a recognition domain for β-galactoside^24^. Gal-3 is ubiquitously expressed on the surface of tumor and metastatic cancer cells, and is involved in multiple processes such as cell to cell, and cell to matrix adhesion as well as tumor growth, and metastasis both *in vitro*, and *in vivo*^25,26^. Since Gal-3 plays an important role in the growth, survival, and spread of cancer cells, it has been proposed as an analytical biomarker of cancer^27^. Modified Citrus Pectin (MCP) has been shown to interact with a GAL-3 domain responsible for carbohydrate recognition, and antagonize the functions related to this domain, such as cell-cell interactions^20,28^. According to studies, MCP counteracts with tumor progression, and metastasis both in *in vitro*, and *in vivo* models, and its GAL-3 inhibitory effect have some roles in sensitizing cancer cells to cytotoxic agents^20^.

In this study, initially, we evaluated cytotoxic activity of both compounds for their ability to decrease the survival of three tumor cell lines. The induction of apoptosis, and cell cycle modifications were studied at cellular and molecular levels to elucidate the effect of CP, and AP exposure on Breast cancer cells. Gal-3 expression alterations and induction of DNA damage in MDA-MB-231 cells in response to CP, and AP treatments were evaluated. We also used MDA-MB-231 multicellular spheroids in a try to further estimate the efficiency of CP cytotoxicity in a more tumor like environment. Moreover, we have demonstrated the interaction of CP, and AP with the DNA extracted from rat hepatocytes. For this purpose, we employed spectroscopic techniques (absorption, emission, and circular dichroism) to ascertain the strength and mode of binding of CP, and AP with DNA.

## Results

### CP and AP inhibited Breast cancer cells growth

**Figure 1:**
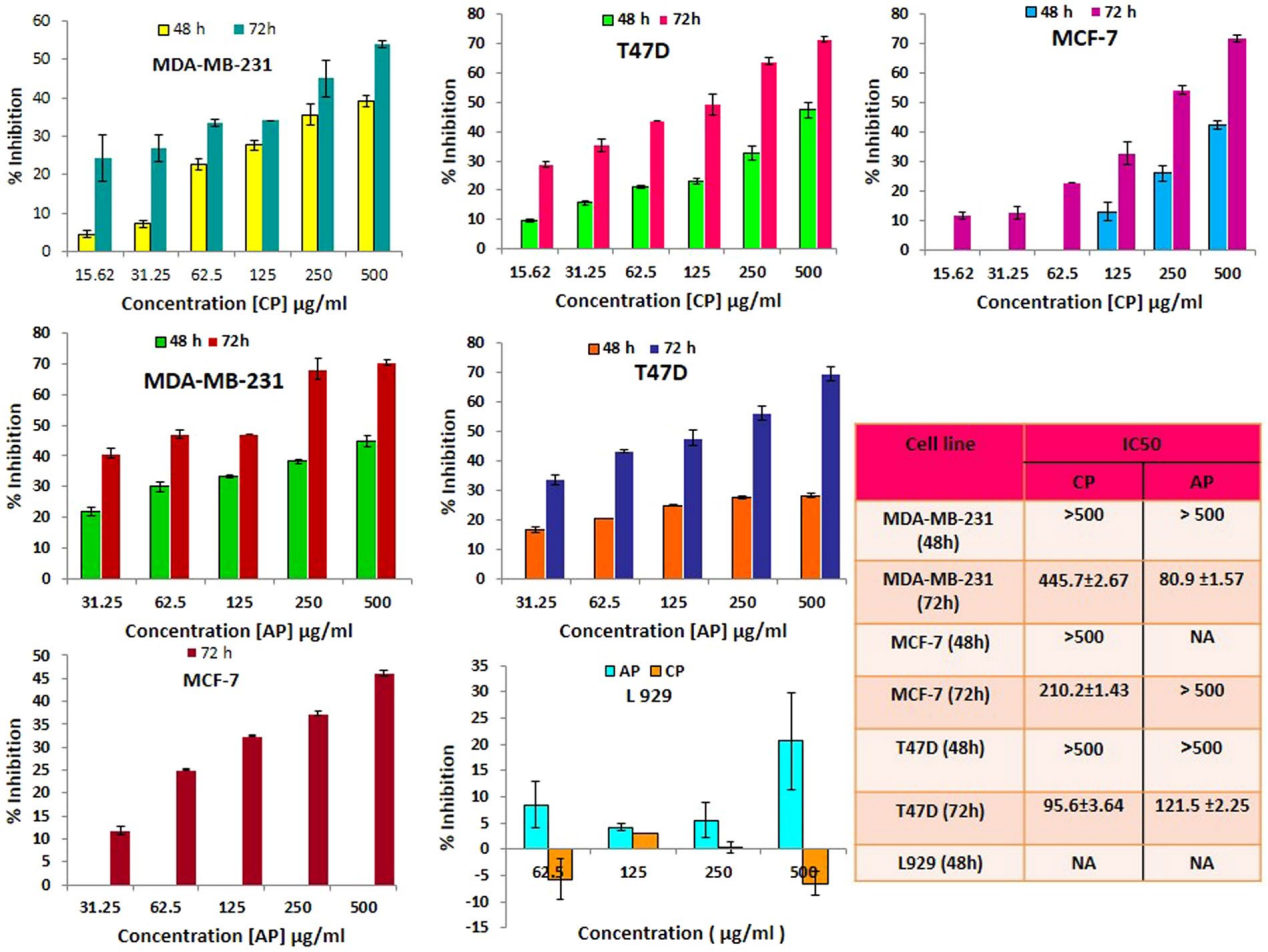
CP and AP inhibit proliferation in human Breast cancer cells. Cytotoxicity of various concentrations of CP, and AP on MCF-7, MDA-MB-231, and T47D Breast cancer cells after 48 and 72 h treatments. Charts are showing CP/AP dose–response. Cells’ viability significantly decreased after incubation with different concentrations of CP and AP (0, 15.62, 31.25, 62.5, 125, 250, 500 μg/ml) for 72 h. The IC50 values are determined by MTT, and data are presented as the mean ± SD of three independent experiments.

Growth inhibition induced by CP, and AP after 48, and 72 h treatments in three human Breast cancer cell lines including MDA-MB-231 (ER−/PR−/HER-2-), MCF-7, and T47D (ER+/PR+, HER-2-), and non-tumorigenic fibroblast L929 cells was evaluated. As it is shown in Fig. 1, treated samples exhibited a dose, and time dependent decline in viability compared to control groups. However, both pectins had much less toxicity against non-tumorigenic L929 cells. As indicated in Fig. 1, CP has reduced viability in T47D, MCF-7, and MDA-MB-231 in 72 h treatment, while no significant inhibition was observed at 48 h, as it is corroborated by their high IC50 values. In addition, incubating cells with 0 to 500 μg/ml of AP for 48 and 72 h exerted high cytotoxicity on MDA-MB-231 and T47D cells, while it did not induce significant cytotoxicity against MCF-7 cells (Fig. 1). As MTT assay results revealed both pectins were cytotoxic toward human Breast cancer cells especially in 72 h treatment, and did not affect non-tumorigenic L929 cells. Furthermore, AP (IC50 = 80.9 ± 1.57 μg/ml) exhibited the highest anti-proliferative activity against MDA-MB-231 cells in 72 h treatment, when compared with MCF-7, and T47D. Since among the examined Breast cancer cell lines, MDA-MB-231 cells are the most invasive cells with the highest proliferation, and metastasis power, we preferred to use this cell line to continue our studies.

### CP and AP cause apoptosis, and DNA fragmentation in MDA-MB-231 cells

**Figure 2:**
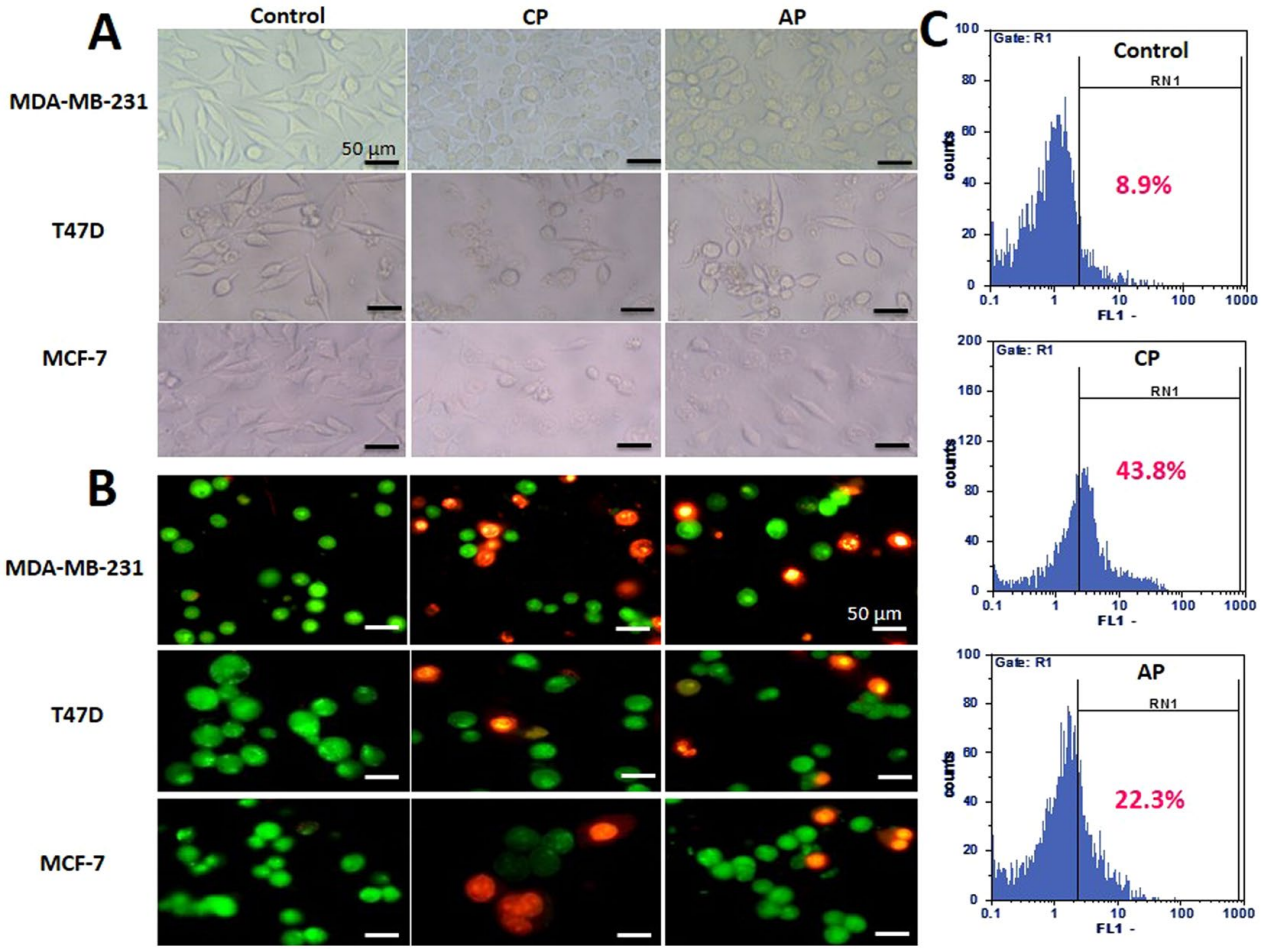
Morphological changes and DNA fragmentation induced by CP and AP. MDA-MB-231, T47D, and MCF-7 cells treated with IC50 concentrations of CP, and AP for 24 h. (**A**) Invert, and (**B**) fluorescence (stained with EB/AO) microscope images showed clear apoptotic morphological changes in the cells. Scale bars represent 50 μm. (**C**) TUNEL assay flow cytometric histograms of MDA-MB-231 cells treated with CP (800 μg/ml), and AP (200 μg/ml) for 4 h, and untreated cells. The right shift of the histogram in CP and AP treated cells compared to untreated control indicates an evident increase in the apoptotic cells population.

**Figure 3:**
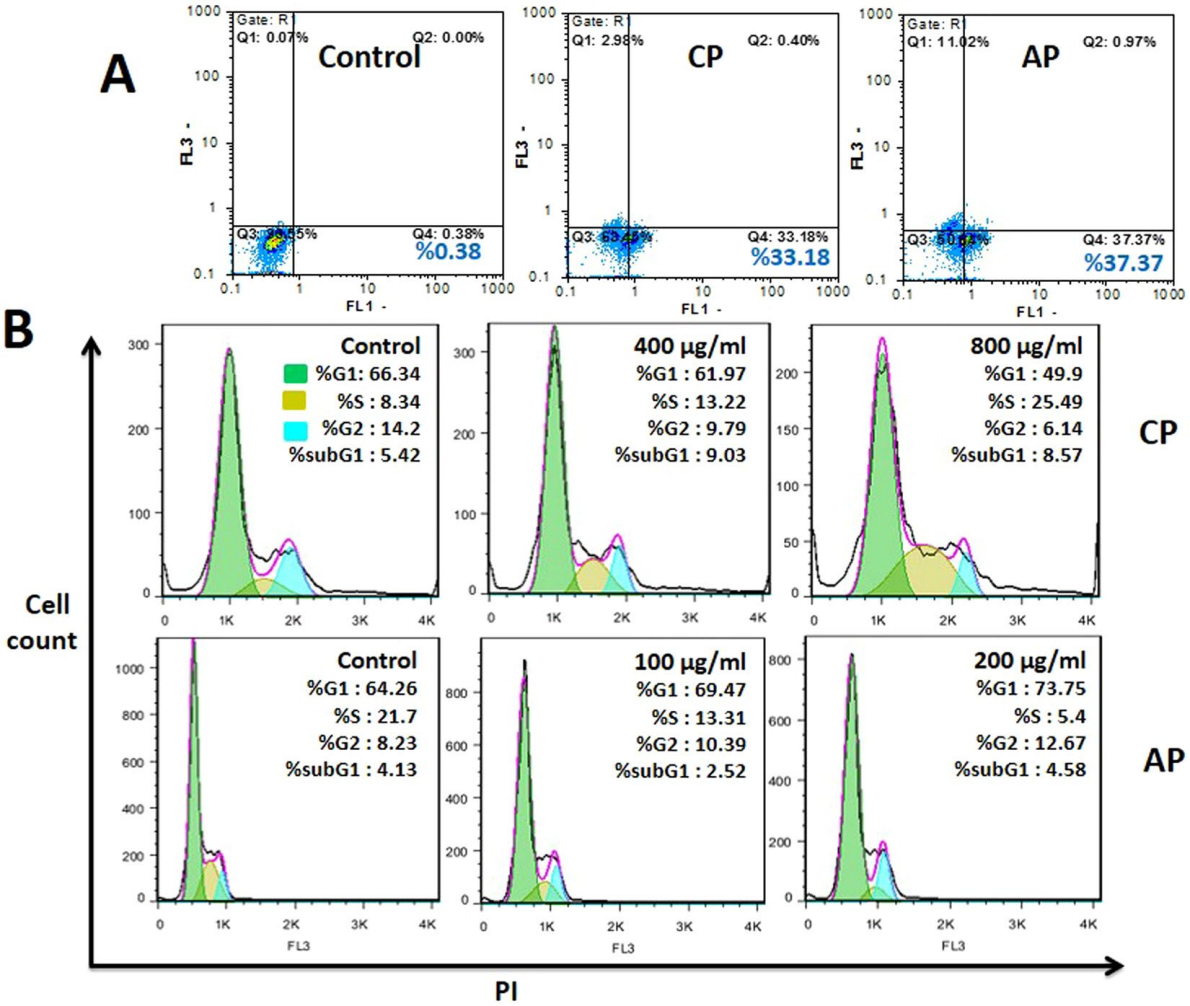
Apoptosis and Cell cycle progression inhibition in CP, and AP treated MDA-MB-231 cells. (**A**) Annexin-V/PI staining Flow cytometry dot plot of MDA-MB-231 cells treated with CP (800 μg/ml), and AP (200 μg/ml) for 4 h. Numbers in the quadrants represent the percentage of MDA-MB-231 cells within each quadrant. (**B**) Cell cycle FACS analysis histograms, and Quantification of treated cells stained with PI. Cells distribution in the G1, S, G2/M, and sub-G1 phases of the cell cycle are shown.

As it was speculated from MTT assay, the decrease in cell viability could be related to the apoptotic effect of the compounds. Therefore, all three Breast cancer cell lines were treated with IC50 concentrations of CP, and AP for 24 h. Initial apoptosis assessment by using invert microscope exhibited vivid morphological alterations associated with apoptosis in treated cells, such as cell shrinkage, irregular morphology, roundness, reduced cell density, and cell membrane blebbing compared with the control (Fig. 2A)^29^. Fluorescent microscope images also showed various extents of apoptosis specific morphological alterations, such as fragmentation and membrane disruption in the nucleus of CP, and AP treated cells as well as chromatin condensation, and increased fluorescence intensity of the nucleus (Fig. 2B), which altogether indicated that both compounds might have promoted apoptotic cell death in all three Breast cancer cell lines. Phosphatidylserine (PS) translocation into the exterior layer of the plasma membrane is one of the first signs of early apoptosis, and due to its excellent binding affinity to Annexin V protein, its detection is a good indicator in estimating apoptosis^30^. To further confirm apoptosis, MDA-MB-231 cells treated with 800 μg/ml (2x IC50) CP, and 200 μg/ml (2x IC50) AP were subjected to Annexin V-FITC apoptosis detection kit. In flow cytometry analysis of untreated cells, the percentages of viable, early apoptotic, late apoptotic, and necrotic cells were 99.55%, 0.38%, 0.00%, and 0.07%, respectively. However, after 4 h of treatment with CP, and AP, the population of early apoptotic cells (Annexin V-positive, PI-negative) increased drastically to 33.18%, and 37.37%, respectively. As it is shown in Fig. 3A, the late apoptotic cells (Annexin V and PI positive) population did not significantly change (0.00% in untreated cells to 0.4% in CP treated, and 0.97% in AP treated cells), While, there was a trivial increase in necrotic cells (Annexin V-negative, PI-positive), ranging from 2.98% in CP treated to 11.02% in AP treated cells. DNA degradation in to oligonucleosome-size fragments is another typical characteristic of cell death, and can be detected by TUNEL assay. After treating MDA-MB-231 cells with 800 μg/ml CP, and 200 μg/ml AP for 4 h, DNA fragmentation was quantified by flow cytometry, and Compared to untreated cells up to 43.8%, and 22.3% apoptosis was observed in treated cells respectively (Fig. 2C).

### AP treated MDA-MB-231 cells inhibit cell cycle at G1, and G2/M phases, but CP at the S phase

CP and AP interventions in the cell cycle were inspected in MDA-MB-231 cells by measuring DNA content via propidium iodide (PI) staining. As Flow cytometry FACS analysis showed, treatment with 400 and 800 μg/ml of CP for 24 h increased cells population in the S phase fraction from 8.3% in controls to 13.2%, and 25.4% in the treated samples, respectively. However, treatment with AP led to the increase of the cell population in the G0/G1 phase from 64.2% to 69.4% (100 μg/ml), and 73.7% (200 μg/ml), furthermore, as it is shown in Fig. 3B, the MDA-MB-231 cells population in the G2/M phase has increased from 8.2% in controls to 10.3%, and 12.6% in the cells treated with 100 and 200 μg/ml of AP, respectively.

### CP and AP decrease Nitric Oxide (NO) production

**Figure 4:**
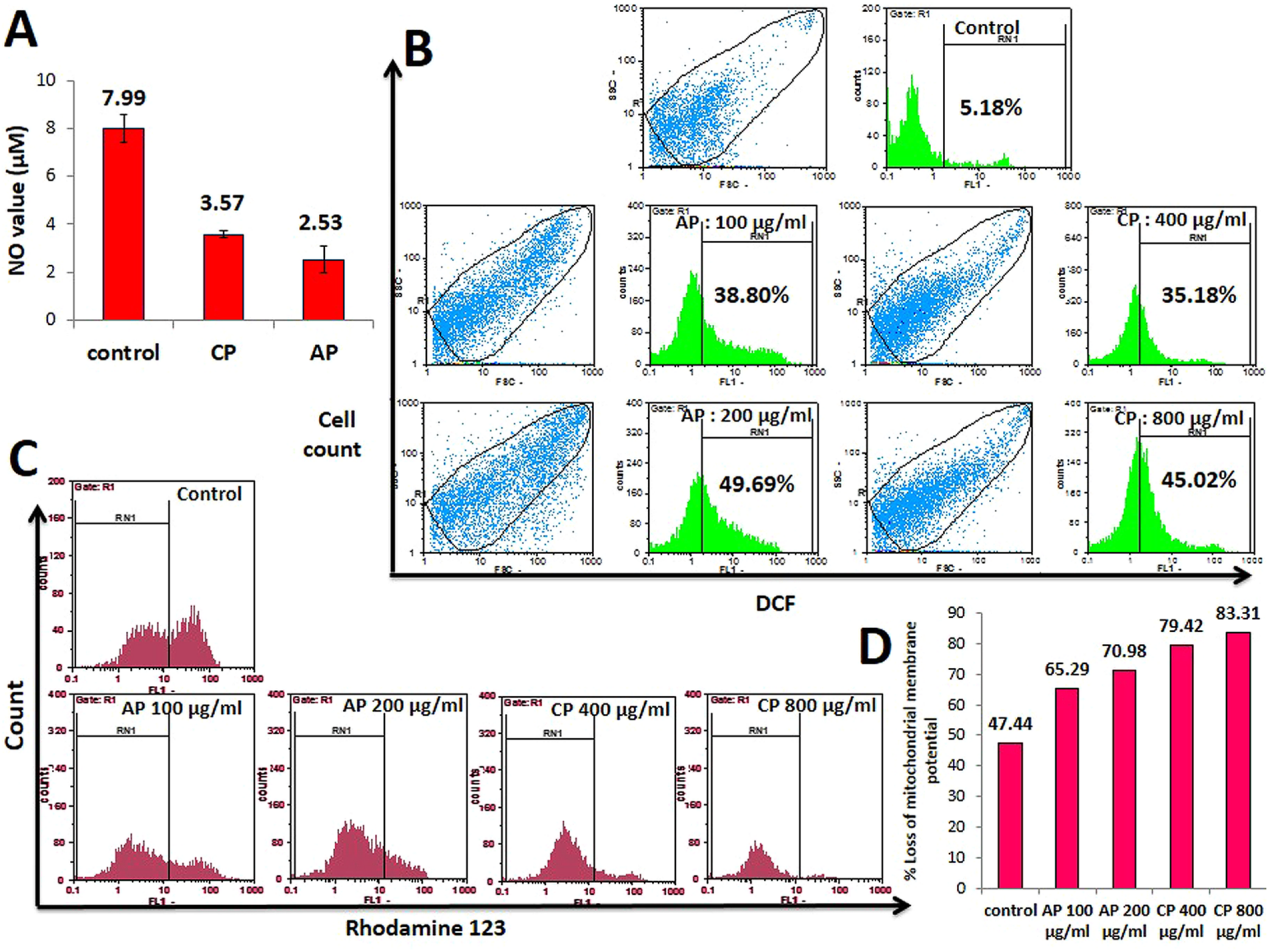
(**A**) Comparison of NO release after treatment with 800 μg/ml CP, and 200 μg/ml AP. The data are expressed as mean ± SD. (**B**) ROS overproduction in CP, and AP treated cells. flow cytometery analysis of DCFH-DA probed MDA-MB-231 cells treated with differente concentrations of CP, and AP for 12 h along with untreated control. (**C**) Mitochondrial membrane depolarization in CP, and AP treated cells. Flow cytometry histograms of Rhodamin123 stained MDA-MB-231 cells treated with CP (400 and 800 μg/ml), and AP (100, and 200 μg/ml) for 12 h. mitochondrial membrane potential changes were measured by comparing the extent of rhodamine123 fluorescent intensity shift in treated, and control cells. (**D**) Diagram shows the percentage of cells with disrupted MMP after CP, and AP treatments.

The amount of NO production in the supernatant of MDA-MB-231 cells after 12 h treatment with CP and AP was determined with Griess reagent. During this period, the NO level decreased remarkably in the cells supernatant compared to the control group (Fig. 4A). Treating cells with 800 μg/ml CP, and 200 μg/ml AP respectively released 3.57 ± 0.15, and 2.53 ± 0.55 μM of NO, respectively, which were significantly lower than the control (7.99 ± 0.6 μM).

### Intracellular ROS overproduction and mitochondrial membrane depolarization are involved in CP and AP induced apoptosis

ROS has a pivotal role in activating cell death pathways. To study the impact of CP, and AP on oxidative stress, intracellular ROS production in CP, and AP treated MDA-MB-231 cells was measured by DAFH-DA probe. As illustrated in Fig. 4B, the ROS levels generated in response to (400, 800 μg/ml) CP, and (100, 200 μg/ml) AP treatments were significantly higher than untreated cells, which indicates that apoptosis is mediated by ROS production, and may have altered the cellular redox status. Excessive ROS production can influence mitochondrial functions, and initiate mitochondrial mediated cell death via disruption of mitochondrial membrane potential (MMP or ΔΨm) in cancer cells^30–32^. ΔΨm was monitored by Rhodamine123 fluorescent probe. Unlike viable cells, mitochondria in apoptotic and necrotic cells cannot sequester Rhodamine123 into aggregate, and diffuse it out in monomeric form, causing a shift in its fluorescence emission. Fig. 4C shows FL-1 plots of Rhodamine123 stained MDA-MB-231 cells, and the percentages of apoptotic cells analyzed by flow cytometer in different treated groups. Results obtained from stained cells, showed a significant dose dependent reduction of MMP in both CP, and AP treated cells. Furthermore, the observed loss of MMP induced by CP was much higher compared to AP. A parallel change in ROS production and MMP value suggests that mitochondria are the major intracellular sources and primary targets of ROS, and CP or AP-mediated ROS overproduction may have caused mitochondrial dysfunction.

### Galectin-3 mRNA expression

As the data obtained from real-time PCR analysis indicated, MDA-MB-231 cells treatment with 800 μg/ml of CP, and 200 μg/ml of AP for 4 h have reduced galectin-3 mRNA expression 0.48 (CI, 0.43–0.55) and 0.78 (CI, 0.73–0.85) folds compared to untreated control, respectively. However, the reduction in CP treated cells was more pronounced, and has reduces galectine-3 expression more than 50%.

### DNA oxidation and DNA strand break in MDA-MB-231 cells induced by CP and AP

**Figure 5:**
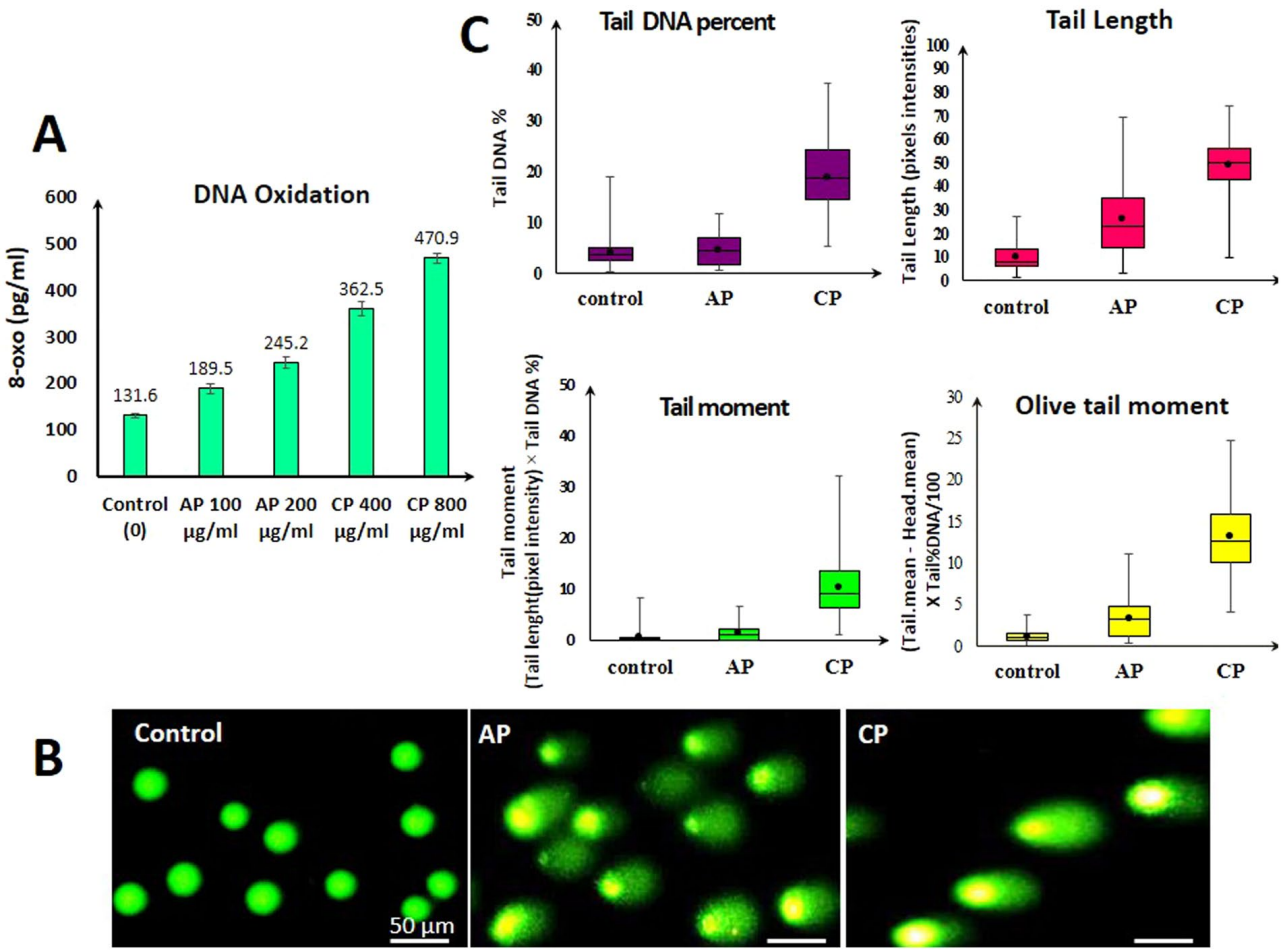
(**A**) Formation of 8-oxo-dG in the genomic DNA of MDA-MB-231 cells after treatment with different concentrations of CP, and AP. (**B**) MDA-MB-231 cells comet assay results. Cells were treated with CP (800 μg/ml), and AP (200 μg/ml) for 2 h, and DNA damage in the form of DNA strand breaks was assessed by comet assay. Fluorescent microscope image of control and treated cells stained with SYBR green dye. Scale bars represent 50 μm. (**C**)The four different comet assay analysis presented as Box and whiskers plots demonstrate increased DNA damage in AP, and in particular CP treated cells.

DNA oxidation is an ongoing event in the everyday life of a cell, and leads to the formation of different lesions including oxidized bases, apurinic/apyrimidinic (AP) sites, and DNA single- and/or double-strand breaks. Compared to other DNA bases, guanine is more prone to DNA oxidation due to its low oxidation potential. 8-hydroxy-2′-deoxyguanosine (8-oxo-dG) is the most common form of oxidized guanine, which is produced by the addition of the hydroxyl radical to the C8 position of the guanine ring, which can then be either oxidized to 8-oxo-dG^33,34^. Increased levels of ROS in CP, and AP treated MDA-MB-231 cells may cause DNA damage. Hence, to explore the effect of both compounds on DNA oxidation, production of 8-oxo-dG was evaluated. As shown in (Fig. 5A), treating MDA-MB-231 cells for 2 h with 400, and 800 μg/ml of CP, and 100 and 200 μg/ml of AP have resulted in increased formation of 8-oxo (362, 470 pg/ml), and (189, 245 pg/ml) respectively in the genomic DNA. Comet assay was used to quantify the extent of DNA damage. CP and AP compounds have induced DNA strand breaks in MDA-MB-231 cells compared to the control. Analysis of approximately 100 cells after 2 h treatment with 2x IC50 concentrations of CP, and AP revealed a significant increase in DNA strand breaks in response to CP, and AP treatments in MDA-MB-231 cells compared to untreated cells. However, CP treatment has caused significantly greater DNA damage to the cells. As shown in Fig. 5B, the migrated cell nucleus with DNA strand breaks in the CP and AP treated cells and the fluorescence intensities of the migrated damaged DNA in the broom-shaped tails of the comets were stronger than in the controls. The comet assay analysis results are presented in Box and whiskers plots (Fig. 5C). These results indicate that the apoptosis induced by CP, and AP is tightly correlated with DNA damage.

### CP inhibits MDA-MB-231 cells via apoptotic cell death at higher concentrations in multicellular spheroids

Monolayer or two-dimensional (2D) cell culture is conventionally used in cytotoxicity assessments. However, this model is unable to reproduce the physiological conditions, and biological barriers that a cytotoxic compound faces to reach cancer cells in a tumor. Therefore, to partially overcome such limitations, three-dimensional (3D) cell cultures or multicellular spheroids, have been devised to better mimic the physiological environment of tumor, and the drugs’ effects on cancer cells^35,36^.

**Figure 6:**
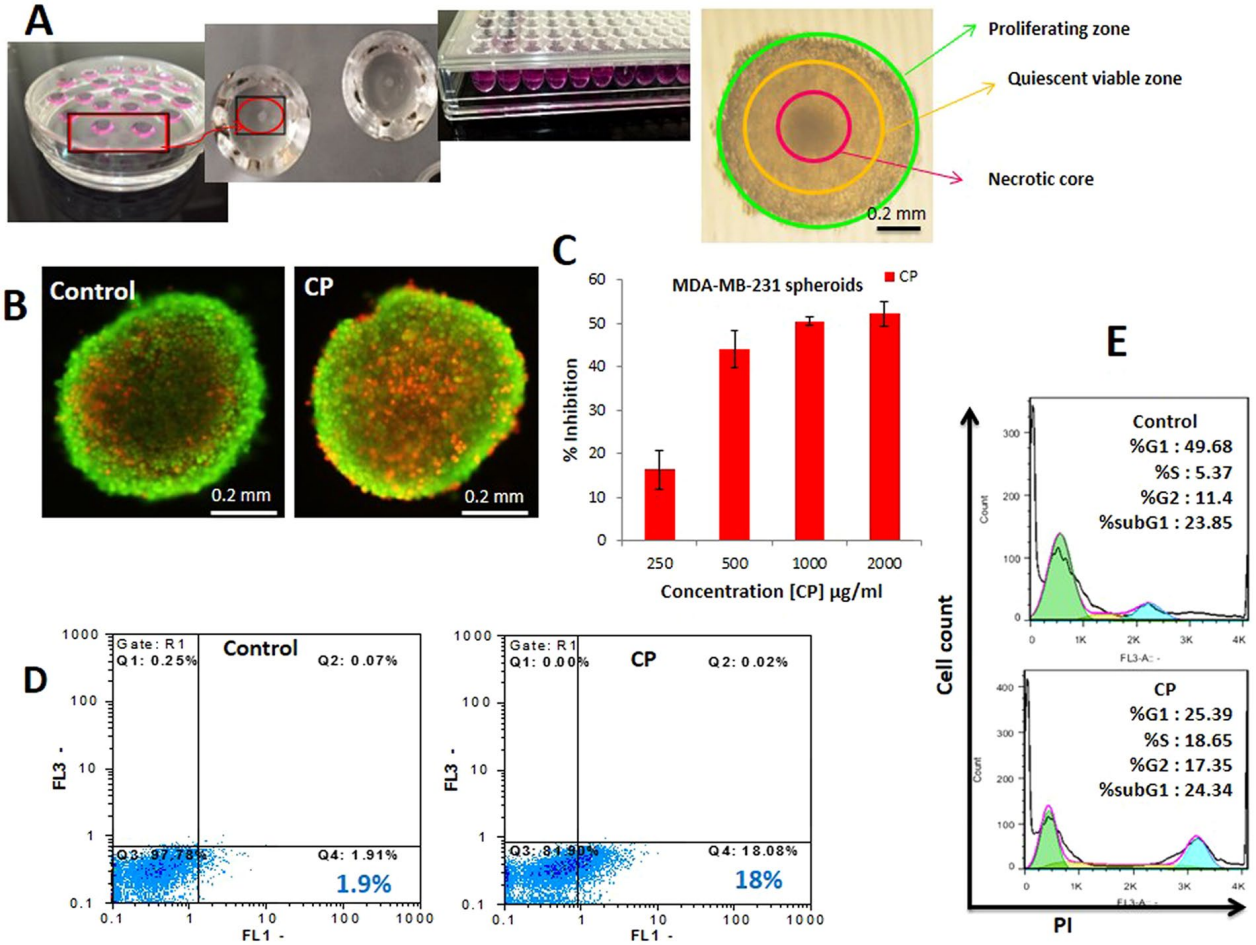
Generation of large MDA-MB-231 cell spheroids, and Evaluation of their cell viability, apoptosis, and cell cycle distribution upon treating with CP. (**A**) An illustration of the 3D cell culture Procedure by HD, and LO techniques to generate large spheroids, and an example of the microscopic characterizations of a large spheroid. (**C**) Concentration-dependent inhibition of MDA-MB-231 cells in the cell spheroids treated with different concentrations of CP. (D1) EB/AO staining of the control and CP treated cell spheroids. The red spots on the surface of the spheroids are EB positive dead cells, which their population has clearly increased in the CP treated spheroid. (D2) The flow cytometry histograms of singled cell control, and CP treated spheroids, stained with AnnexinV-FITC/PI. Apoptotic and necrotic cells were quantified by flow cytometry, and the different subpopulations were defined as Q1, Annexin V−/PI+, necrotic cells; Q2, Annexin V+/PI+. Late apoptotic cells; Q3, Annexin V−/PI−, normal live cells; and Q4, Annexin V+/PI−, Early apoptotic cells. (**E**) The flow cytometry results of the cell cycle distribution were analyzed by the flowJo software, and percentages of cells distribution in different phases of the cell cycle (G0/G1, S, or G2/M phases) were calculated. The Sub-G1 cell population was considered as apoptotic cells.

In this study, large spheroids (starting from about 500 μm in diameter as determined by analysis of spheroid images by ImageJ software), formed by Hanging Drop and Liquid Overlay techniques, were considered as the *in vitro* 3D models of solid tumors. Three distinct zones were observable in these spheroids, a proliferative zone in the outer layer, a quiescent zone in the middle created due to restricted access to oxygen and nutrients, and a necrotic core, which all together resemble the cellular heterogeneity of a solid tumor *in vivo* (Fig. 6A). The percentage of cell death in MDA-MB-231 cell spheroids after CP treatment was 16%, 44%, 50%, and 52% for the wells containing 250, 500, 1000 and 2000 μg/ml CP, respectively (Fig. 6C). Our results indicated that MDA-MB-231 cells 3D cell culture was 2.5 times more resistant to CP treatment (IC50 = 1200 μg/ml) than its corresponding 2D cell culture. The expected increase in the dose response in MDA-MB-231 cells in 3D cell culture reflected the *in vivo-like* microenvironment created in the cell spheroids, which is generally more resistant to anti-cancer drugs than 2D cell cultures. As analysis of cell spheroid images by ImageJ software revealed, a significant 15 ± 3.2 percent reduction in the volume of the CP treated spheroids compared to controls was observed. Apoptosis was also estimated in CP treated spheroids via EB/AO, and AnnexinV-FITC/PI staining. In EB/AO staining, microscopic observations revealed a clear alteration in the dead cells population of the external proliferating, and the internal quiescent zones of the CP treated cell spheroids (Fig. 6B). To further strengthen the data obtained from EB/AO staining, AnnexinV-FITC/PI staining was also employed. As flow cytometry analysis revealed (Fig. 6D), CP in its IC50 concentration has strongly inhibited the proliferation of MDA-MB-231 cells in the treated spheroids. The percentage of apoptotic cells rose from 1.9% in untreated controls to 18% in CP treated spheroids. This data suggests that CP has inhibited MDA-MB-231 cells growth in spheroids through the induction of apoptosis. The cell cycle distribution was also evaluated by FACS analysis and showed that upon treatment of MDA-MB-231 cell spheroids with CP IC50 for 24 h, cells population in the S and G2 phases of the cell cycle has increased significantly. The population of S phase arrested cells was 5.37% and 18.65% respectively in untreated controls, and CP treated spheroids, which showed a 3.5 folds increase in CP treated spheroids (Fig. 6E). G2/M phase arrested cells have also increased from 11.4% in controls to 17.35% in CP treated spheroids.

### Absorption spectra of the Interaction between DNA and CP/AP

**Figure 7:**
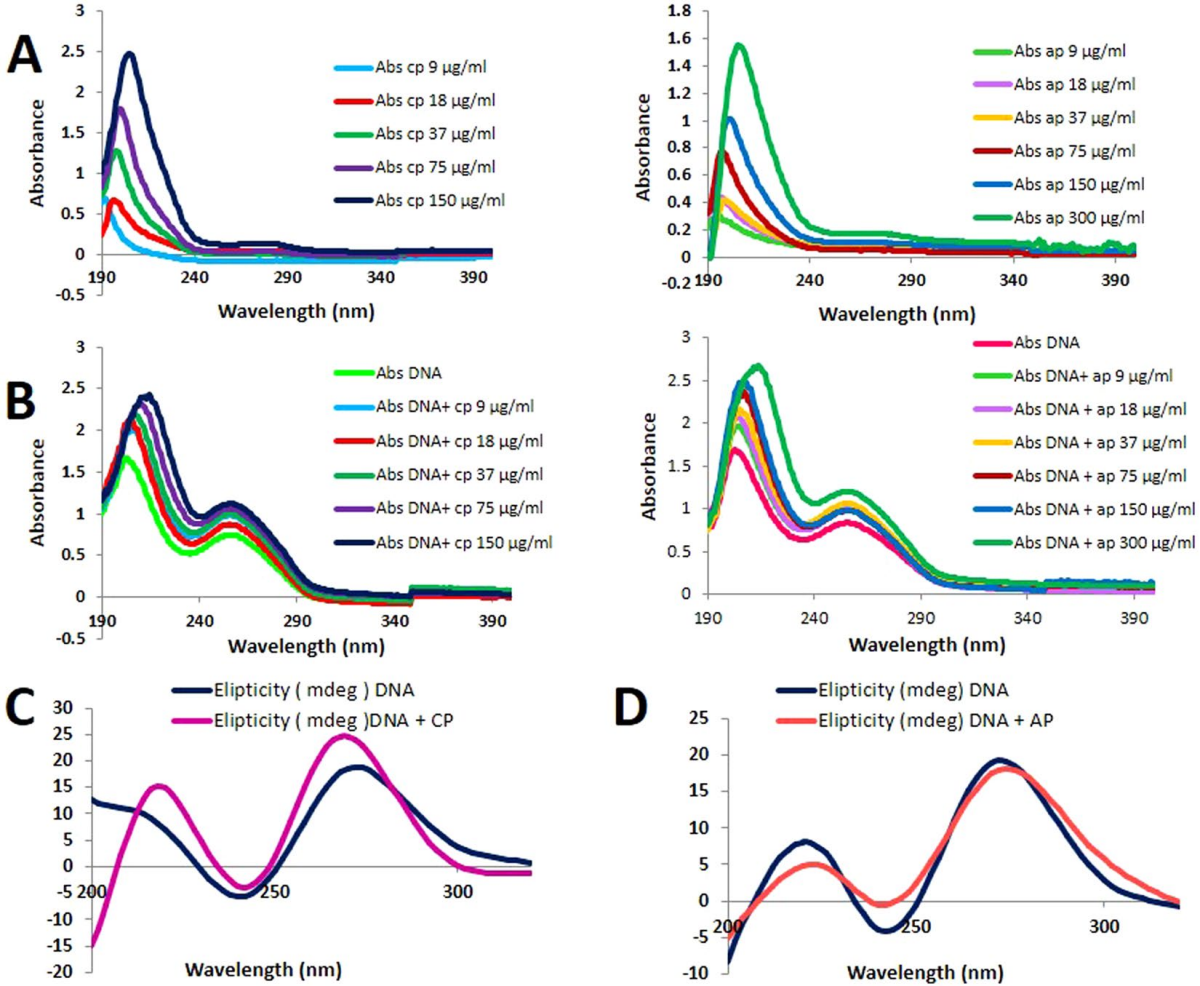
(**A**) UV absorption spectra of different concentrations of CP and AP. (**B**) UV absorption spectra of DNA (50 μg/ml) upon addition of increasing amount of CP (0–150 μg/ml) and AP (0–300 μg/ml) (**C**,**D**) Circular dichroism spectra of DNA (100 μg/ml) in the presence of 75 μg/ml of CP, and AP (pH 7.4, room temperature).

Absorption spectroscopy is one of the most useful and effective techniques in DNA-drug interaction studies. This technique allows recognizing the existence of interactions between molecules and DNA, as well as specifying the mode of interaction by utilizing the perturbations in the spectral observation^37^. Binding of different compounds to DNA may create a strong stacking interaction between the compounds, and the base pairs of the DNA, and may cause the absorbance spectrum shows some degrees of hypo- or hyperchromism. Fig. 7 shows the spectra of unbound DNA, CP, AP, and the DNA-CP/AP complexes in phosphate buffer (0.1 M, pH = 7.4). In the UV region DNA displays two peaks, one at 260 nm (indole groups), and the other at 210 nm (sugar-phosphate groups), while, CP, and AP have no absorption in these areas. As shown in Fig. 7B, upon addition of increasing amounts of CP or AP to the DNA solution, a remarkable enhancement in the absorption of DNA was observed, and hyperchromism with small shift (red shift) in the position of 260 maximum absorption peak, and a significant shift in the 210 nm (red shift) absorption peak also took place, which altogether suggest that a complex between CP/AP, and DNA has been formed.

### DNA and CP/AP interaction characterization by Ethidium Bromide displacement assay

**Figure 8:**
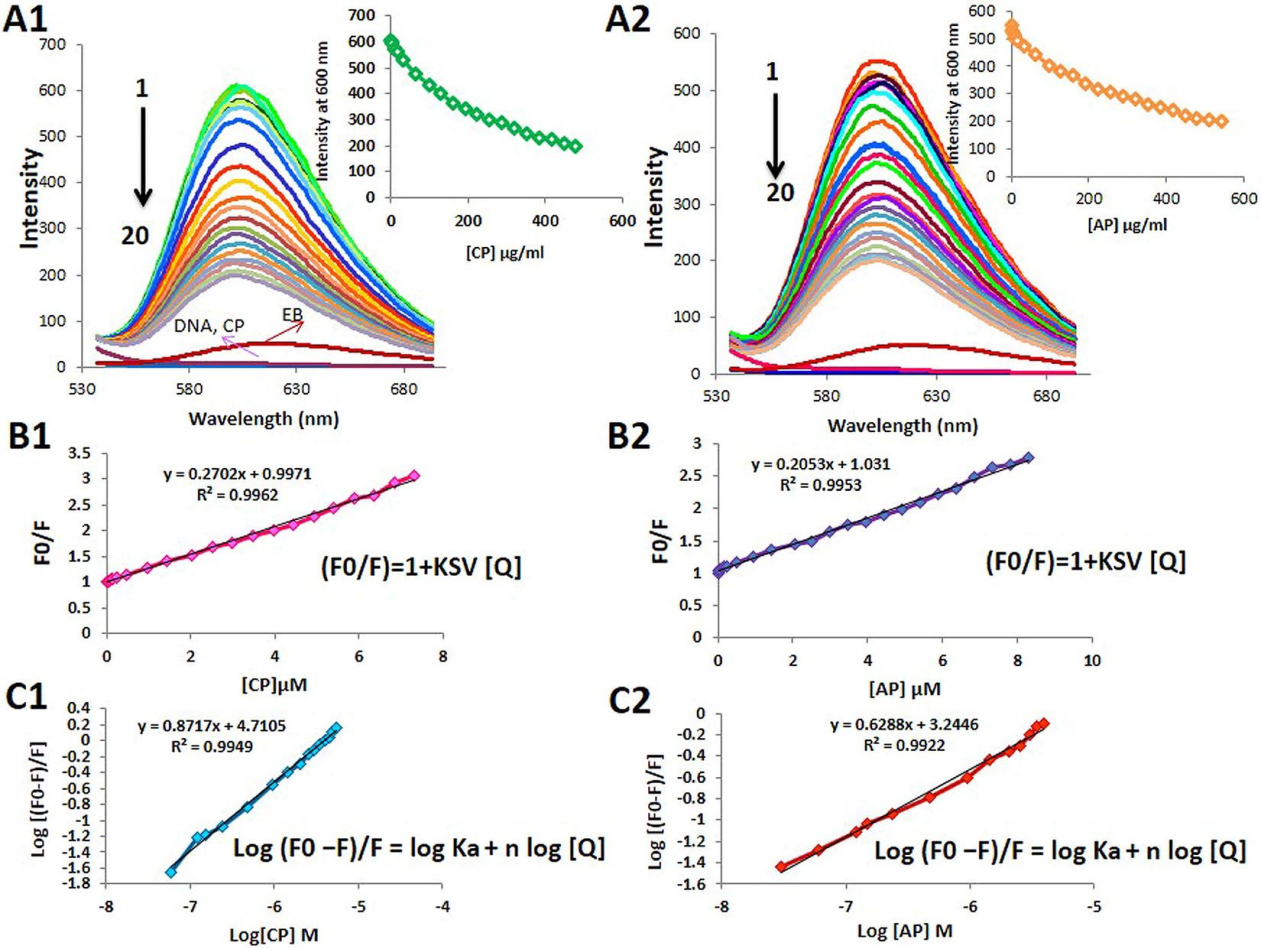
(**A1** and **A2**) Fluorescence spectra of DNA-EB in the presence of increasing amounts of CP, and AP (pH = 7.4, temp = 310 K, λ_ex_ = 500 nm, λ_em_ = 530–700 nm, DNA = 50 μg/ml, EB = 2.6 μM, CP = 0–7.3 μM and AP = 0–8 μM). Insets are the fluorescence of EB (at λ = 600 nm) by adding various concentrations of CP, and AP. (**B1** and **B2**) The fluorescence quenching effect of adding increasing amounts of CP, and AP to the DNA-EB system is illustrated in the Stern-Volmer plot. (**C1** and **C2**) Modified Stern-Volmer Plot (log ((F−F)/F) = log Ka + n log [Q]) for calculating the binding constant (Ka), and number of binding sites (n). F0 and F are the fluorescence intensities before, and after the addition of the CP, and AP, respectively.

The heterocyclic moieties of ethidium bromide (EB) molecules are able to stack between the base pairs of the DNA double helix, and act as fluorescent tags, which make the DNA-EB complex highly fluorescent. This complex fluoresce upon excitation at 500 nm, and can be used as a reliable system to characterize the interaction between small molecules and DNA^37,38^. Following the addition of DNA-interactive molecules to the DNA-EB system, EB is forced to be replaced by the molecules, and a decrease in the fluorescence occurs. The degree of dye displacement, and the subsequent fluorescence reduction provides an indirect measure of the relative strength of the interaction between DNA and the binding compound. Figure 8A–C, summarizes the binding of CP, and AP to the rat hepatocyte DNA at pH = 7.4. Upon addition of increasing amounts CP, and AP to the DNA-EB solution EB rapidly displaced in the complex until reaching a saturation point. Consequently, the fluorescence intensity of DNA-EB at around 600 nm gradually decreased, however, No significant shift in the maximum emission wavelength of DNA-EB was observed. The results indicated that, both compounds could quench the fluorescence of DNA-EB. Quenching processes are often categorized into dynamic and static quenching. Dynamic or collisional quenching is when the excited fluorophore makes contact with the quencher, in which non-radiative transition to the ground state took place. Whereas, in static quenching a stable non-fluorescence complex forms between the fluorophore and the quencher. Dynamic and static quenching can be distinguished by the Stern-Volmer equation (Fig. 8B1 and B2). As it is illustrated in Fig. 8B1 and B2, Stern-Volmer plots of both compounds are linear, suggesting that the dynamic quenching process might have occurred. Therefore, in order to calculate the binding constant (Ka), and the number of binding sites (n) modified Stern-Volmer equation was used (Fig. 8C1 and C2). As it is shown in Fig. 8C1 and C2, the plots were linear, and the binding constant (Ka) for the interaction between DNA and CP was 5.1 × 10^4^ M^−1^, which indicated that CP had a high affinity towards dsDNA, and the binding mode should be intercalation. The calculated number of binding sites (n) for the CP-DNA complex was also 0.87. In the case of DNA-AP interaction the calculated Ka and n value were 1.7 × 10^3^ M^−1^, and 0.62, respectively. The DNA-AP binding constant was lower than the reported binding constant of classical intercalation, which indicated a non-intercalative binding mode.

### Circular Dichroism (CD) Studies

CD spectroscopy is able to detect DNA conformational changes in the presence of DNA binding molecules^39^. In the CD spectroscopy, the unbound B-DNA is characterized by a negative band at 245 nm due to its right handed helicity, and a positive band at 275 nm due to base stacking. Small molecules electrostatic interactions, or groove binding do not perturb base stacking, and helicity bands significantly. In contrast, intercalation drastically alters the intensities of these two bands in favor of more stabilizing the B-DNA conformation^11,40^. The CD spectra of CP/AP- DNA complexes are shown in Fig. 6C and D. The free hepatocyte DNA exhibited the two conservative CD bands, however, at the presence of CP (75 μg/ml) the intensities of both negative and positive bands increased significantly, and accompanied by a vivid blue shift in the positive band (Fig. 7C). This indicates that CP predominantly intercalates between the base pairs of the DNA, and enhances the base stacking, and helicity. On the other hand, upon treating with 75 μg/ml of AP, the CD spectrum of the DNA exhibited an increase in the negative band intensity, and a low perturbation on the positive band (base stacking) without any significant shift (Fig. 7D), which reveals that the interaction between AP, and DNA is via partial intercalation, and groove binding.

## Discussion

Diet, and nutrition have been considered as effective strategies in the prevention of Breast cancer^41^. Moreover, numerous studies indicate that a quite diverse category of dietary natural components can slow down Breast cancer progression at different stages through the induction of apoptosis, proliferation, angiogenesis, and metastasis inhibition as well as sensitization of Breast cancer cells to chemotherapy. Hence, natural dietary ingredients can serve not only in the prevention of Breast cancer, but also their bioactive components can be considered as possible antitumor drugs^42^.

One of the major obstacles in cancer therapy is multidrug resistance which causes the repeated reoccurrence of tumor at the primary site of incidence. To tackle this problem, recently various studies reported the anticancer potential of herbal extracts with minimal toxicity to normal cells. Taking these facts into consideration, two plant products (CP and AP) were selected based on their therapeutic potentials.

Pectin is a heteropolysaccharide found naturally in the cell wall of fruits, and vegetables with beneficial effects on human health^43^. In industrial scale, the main sources of pectin are apple and citrus fruits. Pectin’s biological properties depends largely on its molecular weight, degree of esterification, and viscosity^20,44^. Pectin’s function as an anticancer agent depends on its ability to induce apoptosis in cancer cells, and in different studies CP, and AP have been modified in different conditions to improve this ability. Previous studies have shown that CP, and AP can induce apoptosis in cancer cells, however, their mechanism of action has not been understood very well. In this study, we have investigated whether CP and AP in their intact forms are able to induce apoptosis in MDA-MB-231 cells, blocking galectin-3, and interact with genomic DNA. To examine this, the cytotoxicity of CP, and AP at different concentrations was assessed on T47D, MDA-MB-231, and MCF-7 cancer cells. As MTT assay results indicated, both compounds were capable of increasing cell death in Breast cancer cells in a dose, and time-dependent manner. A higher cytotoxicity was obtained when MDA-MB-231 cells were treated with AP (IC50 = 80 μg/ml), and T47D cells with CP (IC50 = 95 μg/ml) for 72 h, which also caused a dose-dependent reduction in the cells viability. Furthermore, apoptosis specific morphological alterations, and DNA fragmentation, were observed in treated cells as light and fluorescent microscopy, and tunnel assay indicated, which supported previous studies showing the antiproliferative, and apoptotic effects of pectin in Breast cancer cells^22^. It worth noting that, treating L929 normal cells with CP, and AP did not induce any cytotoxicity. The anti-proliferative effect of both pectins on malignant cells has been attributed to various mechanisms. Delphi *et al*. have reported that apple Pectic-oligosaccharides cause sub-G1 cell cycle arrest, and induce caspase dependent apoptosis in MDA-MB-231 cells^45^. Moreover, Sun *et al*. have shown that, Apple extract arrests MCF-7 cells at the G1 phase of the cell cycle by reducing cyclin D1, and Cdk4 proteins expression^46^. Delphi *et al*. have also demonstrated that the apple pectic acids are able to induce death in 4T1 Breast cancer cells via apoptosis *in vitro*, and cause overexpression of p53, and subsequent inhibition of tumor metastasis in BALB/c mice *in vivo*^22^. Lemon citrus extract have also shown to upregulate bax, and caspase-3 genes expression, downregulate bcl-2 gene expression, and consequently, induce apoptotic cell death in MCF-7 Breast cancer cells^47^. Anticancer effect often is mediated by the inhibition of proliferation, and cell cycle arrest. As our flow cytometric analysis of cell cycle revealed, the antiproliferative effect of AP was accompanied by cell cycle arrest at the G1, and G2/M phases in MDA-MB-231 cells, whereas, CP arrested MDA-MB-231 cells in the S phase. We also investigated the gal-3 mRNA expression changes in CP, and AP treated MDA-MB-231 cells. Galectin-3 is over expressed in Breast cancer cells, and antagonize the cytotoxic effects of different drugs such as staurosporine, and cisplatin as well as stimuli like radiation, and nitric oxide by inhibiting the intrinsic apoptotic pathway^48,49^. Furthermore, Galectin-3 is responsible for loss of cell adhesion, and increased metastasis in Breast cancer tumors^50^. Although the exact anti apoptotic mechanism of Gal-3 has remained to be elucidated, it is suggested that it functions via maintaining mitochondrial integrity, and caspase inhibition^51,52^. Previous studies suggested that galectin-3 may be a potent inhibitor of mitochondrial mediated intrinsic apoptosis in Breast epithelial cells^26^. Among the galectin-3 inhibitors, many researchers have focused on pectins due to their low toxicity. Furthermore, it has been demonstrated that the pectins antiproliferative activity is in direct relation with the amount of galectin-3 expression on the surface of the cancer cells^20^. The galactose residues in AP, and particularly CP, similar to many tumor-associated antigens (TAAs) are responsible for interaction with Gal-3; however, in contrast to TAAs their binding to Gal-3 exerts antiproliferative effects and could be one of the possible mechanisms that both compounds inhibit tumorigenesis. Many previous studies have focused on Modified Citrus Pectin (MCP) as Gal-3 inhibitor with the ability of binding to Gal-3 as a ligand and claiming that due to limited solubility in water CP and AP in their naive form are unable to interact with Gal-3. However, the latest studies have reported that Pectin is a water-soluble dietary fiber^53^, and exhibit biological activities, ranging from anti-inflammatory, and anti-microbial activities to cardiovascular protective role^54^. A representative compound GCS-100, is a modified polysaccharide extracted from the peel and pulp of citrus fruits with high pH and temperature treatment, and is a Gal-3 antagonist that inhibits myeloma cell growth *in vitro*^55^. In addition, RN1 is another homogeneous neutral polysaccharide, purified from the flower of Panax notoginseng binds to Gal-3 and significantly suppresses its mRNA expression and downregulates the protein expression of Gal-3 in a time-dependent manner^56^. In the current study, we have demonstrated that galectin-3 mRNA expression was reduced in MDA-MB-231 cells treated with CP, and AP in their naive form. Kolatsi-Joannou *et al*. have also reported that MCP did not alter galectin-3 mRNA levels initially (at 2 days), But there was a statistically significant reduction at day 14^57^. CP binding to gal-3 could be one of the possible mechanisms of CP inhibiting tumorigenesis because many tumor associated antigens bind to gal-3 through their galactose residues to promote the growth and progression of tumor.

Previous studies have suggested that nitric oxide overproduction mediates pectin induced apoptosis^58,59^, but quite the contrary, here we have shown that treating MDA-MB-231 cells with CP, and AP have reduced NO production significantly, and instead have caused a rapid increase in ROS production and loss of mitochondrial membrane potential, which provides new insights into the mechanism of cell death by CP, and AP. ROS plays various physiological functions, and has both pro- and anti-apoptotic roles according to its concentration. ROS overproduction has been correlated with apoptosis^60–63^, and since the ROS level has increased significantly in the presence of CP, and AP, it can be concluded that both compounds are capable of increasing the mitochondria membrane permeability in MDA-MB-231 cells, and inducing apoptosis via ROS accumulation. On the other hand, mitochondria are also responsible for intracellular ROS production and apoptosis and suggesting that ROS might be involved in the cytotoxicity induced by CP and AP in MDA-MB-231 cells. Alteration in mitochondrial membrane permeability leads to the formation of mitochondrial permeability transition pores (MPTP) which facilitates unchecked inflow of solutes and free radicals inside the cell. This uncontrolled inflow causes increase of osmotic pressure inside the inner mitochondrial complex and thus finally leads to the overall collapse of the mitochondrial membrane which is considered as the initial step for the activation of the intrinsic cell death pathways^64^.

Consequent analysis of cell cycle arrest, nuclear condensation staining and early apoptotic observation via Annexin/PI staining, DNA damage analysis through fragmentation and comet assay confirmed the apoptotic cell death process. ROS overload also prompts oxidative damage to the cell’s biomacromolecules, which culminates in the cells dysfunction and death. ROS overproduction causes DNA damage via oxidation and DNA strand brakes^61,65^. The level of 8-oxo-dG which is the major product of DNA oxidation by reactive oxygen species, increased significantly after CP, and AP treatment in MDA-MB-231 cells. AP, and in particular CP have also increased DNA strand breaks as it was observed in the comet assay. The formation of DNA breaks clearly indicated the role of CP in causing structural damage to the DNA double helical structure by increased DNA oxidation in the CP treated cells with respect to untreated group.

According to our findings, both CP and AP had considerable cytotoxicity towards the examined Breast cancer cell lines. Initially, as MTT assay results indicated AP exhibited more cytotoxicity against MDA-MB-231 cells, however, in the course of our study, we concluded that CP is more efficient in inducing apoptosis in MDA-MB-231 cells. Some of very evident features of apoptosis such as DNA fragmentation, and mitochondrial membrane depolarization were more pronounced in CP treated MDA-MB-231 cells as TUNEL assay, and ΔΨm analysis showed, respectively. More importantly, the extent of DNA damage in the form of DNA oxidation, and DNA strand breaks was significantly higher in CP treated cells. In addition as it will be discussed below, the mode of CP-DNA interaction was intercalation, which in terms of DNA-drug binding is more favorable. Regarding these results, in order to assess the cytotoxicity of CP in physiological conditions that is more similar to tumor environment, we created compact MDA-MB-231 multicellular spheroids in the cell culture. Our data indicated that MDA-MB-231 cells were still sensitive, but, more resistant to CP treatment when grown in 3D (IC50 = 1200 μg/ml). Our data indicates that CP inhibits MDA-MB-231 spheroids cells’ proliferation, and induces apoptosis by arresting cell cycle progression at the S⁄G2 phase.

Biologically important small molecules like steroids and carcinogens or some effective antitumor drugs such as anthracyclines predominantly interact with DNA within the cells. Hence, searching for DNA binding small molecules has attracted attentions, since they can provide a suitable platform, and useful clues for designing more efficient DNA targeting drugs^40^. In general, noncovalent binding of small molecules to double-helix DNA is categorized into three groups: (i) electrostatic interaction (ii) groove binding, and (iii) intercalation between the base pairs. Intercalative, and groove binding agents interacts with DNA within the grooves of the double helix, whereas, the electrostatic binding can take place outside the grooves. Intercalative binding however, is the most effective mode of DNA-drug interaction in terms of the antitumor activity of the compound^9,11^. Our experiments demonstrated that CP, and AP, in their naïve forms, exhibit significant *in vitro* cytotoxicity against Breast cancer cells in a dose- and time-dependent manner, and for the first time in this study, we showed that CP, and AP are able to interact with double-helix DNA. There are three classes of natural or organic synthetic polymers by charge; cationic, anionic and neutral polymers. Cationic polymers are well documented in many studies for binding to anionic DNA to provide a controlled release of DNA polyplexes. Some neutral polymers have shown the ability to interact or bind to DNA. There are many non-ionic neutral polymers including polyvinylpyrrolidone (PVP), polyvinylpolypyrrolidone (PVPP) and poly (4-vinylpyridine-N-oxide) which are able to interact with DNA. For example, the interaction between DNA and PVP is based on hydrogen bonding with the base-pair in the DNA major groove that may lead to improved cellular uptake of DNA and enhance the transfection efficiency^66^. In the present study, we attempted to investigate CP and AP neutral polymers for their interaction with the dsDNA. Among the techniques frequently used to detect the interaction between DNA and small molecules, we employed UV-vis absorption, fluorescence, and circular dichroism (CD) spectroscopy to investigate the binding mode of CP, and AP to DNA. The UV-vis studies revealed that the absorption peak of DNA at 260 nm increases gradually with increasing concentration of both CP, and AP, which suggested that the binding between the two compounds and DNA, had occurred. Fluorescence and CD studies also indicated that CP binds DNA via intercalation, whereas, AP can binds DNA via partial intercalation and groove binding. The binding constants (Ka) of the CP, and AP with DNA were 5.1 × 10^4^, and 1.7 × 10^3^ M^−1^ at 310 K, respectively.

In conclusion, recent studies on pectins’ anti-cancer properties mostly have focused on modified pectins originated from citrus, while, our results indicated that the pectic polysaccharides from apple and citrus in their naive forms are also capable of inducing apoptotic death in cancer cells. Furthermore, we have suggested that ROS over production via increasing the mitochondria membrane permeability might be the underlying apoptosis mechanism of both compounds. ROS accumulation also has caused oxidation and strands breaks in the DNA of CP and AP treated breast cancer cells. Moreover, our drug-DNA interaction studies also indicated that CP binds DNA via intercalation, whereas, AP can binds DNA via partial intercalation and groove binding. These data suggest CP vs AP can have a potential role in prevention and treatment of human Breast cancer.

## Materials and Methods

### Cell cultures, treatments, and cell survival evaluations

MCF-7, T47D, and MDA-MB-231 human Breast cancer cell lines, and normal fibroblast “L929” cell line were obtained from pasture cell bank in Iran. Cells were grown in RPMI 1640 (Gibco) media supplemented with 1% streptomycin/penicillin, and 10% fetal bovine serum (Gibco). All cell lines were grown in a humidified atmosphere at 37 °C with 5% CO_2_. A stock solution of 1% (W/V) CP or AP was freshly prepared for each experiment. In detail, One gram of both pectins dissolved in 10 ml of distilled water under continuous stirring at 70 °C until a homogenous solution was obtained. The solution was sonicated using an ultrasonic system at 140 W for 5 min at 30 °C, and stored at 4 °C. After adjusting the pH to 7.4, the pectin solutions were diluted further with the culture media until achieving the desired concentration. It should be noted that CP, and AP were soluble in pure water and were stable at room temperature at neutral pH.

The cellular viability of CP and AP treated MDA-MB-231, MCF-7, T47D and L929 cells was evaluated by MTT [3-(4,5-dimethylthiazol-2-yl)-2, 5-diphenyltetrazolium Bromide] (Sigma-Aldrich) assay and 50% inhibitory concentration (IC50) was calculated for each cell line. Briefly, all cells were seeded in 96-well plates to a density of about 1.4 × 10^4^ cells/well and supplemented with 200 μl medium per well. 24 h later, cells were treated with 0–500 μg/ml of CP and AP for 48 and 72 h at 37 °C in 5% CO_2_. After treatment, the medium was replaced with 200 μl freshly prepared MTT solution (0.5 mg/ml in phosphate-buffered saline) and incubated at 37 °C for 4 h. Subsequently, MTT solution was discarded, and 100 μl/well DMSO was added to solubilize the formazan crystals and the absorbance was measured at 492 nm using an ELISA reader (Model wave xs2, BioTek, USA). The concentration of pectins with 50% cell growth inhibition was reported as the IC50 value.

### Microscopic observation of apoptosis in Breast cancer cells

Apoptosis specific morphological alteration in treated cells was observed, and AO/EB viability staining assay was performed to evaluate the ratio of Live/Dead cells. In brief, 2.1 × 10^6^ MDA-MB-231, T47D and MCF-7 cells were seeded in 6 well plates and after 24 h they were treated with CP and AP IC50 for 24 h. After incubation, both treated and untreated cells were observed under invert light microscope and imaged. Subsequently, cells were harvested, centrifuged to collect the cells, and resuspended in PBS. An EB/AO staining solution was then added to the suspension, and the stained cells were visualized and imaged by a fluorescence microscope (Axioskop 2plus, Ziess, Germany). Green cells with intact and condensed nuclei were identified as viable and early apoptotic cells, respectively. While, fluorescently red cells were assigned as late apoptotic and necrotic cells. Twenty randomly selected areas were imaged on each slide to ensure the acquired data were representative.

### Detection of apoptosis in MDA-MB-231 cells by Annexin V-FITC /PI staining and TUNEL assay

To further confirm, and also quantify the percentage of cells undergoing apoptosis, Annexin V–FITC /PI staining and TUNEL Assay were implemented. The primary experimental procedure for both assays was to seed 3.5 × 10^5^ cells/well MDA-MB-231 cells in six-well plates, and treating them 24 h later either with CP (800 μg/ml) or AP (200 μg/ml) for 4 h. After treatment, cells were collected, washed with PBS, resuspended in binding buffer and stained with Annexin V–FITC and PI according to the manufacturer’s protocol. Flow cytometry (Partec PAS, Germany) analysis was performed to identifying the distribution of differentially labeled cells, and the results were analyzed with Flomax software. DNA fragmentation in CP and AP treated MDA-MB-231 cells was detected by using *In situ* cell death detection kit, fluorescein (Roche) which is based on Terminal deoxyribonucleotidyl transferase dUTP nick end labeling (TUNEL) method. In brief, after treatment, cells were washed with PBS, and recollected. The pellets were resuspended in a fixing solution of 4% paraformaldehyde in PBS (pH 7.4) and incubated for 1 h at room temperature with agitation. Afterwards, the fixed cells were permeabilized with 1% Triton X100 in a 0.1% sodium citrate solution. Permeabilization solution was then discarded after centrifugation and cells were washing with PBS twice. Subsequently, the staining solution was added to the pellets and incubated for 1 h at 37 °C in the dark. The Staining solution contained TdT enzyme and its substrate fluorescein conjugated dUTP for the repair of the nicked 3′-OH DNA ends. For data collection about 10,000 cells were counted on the flow cytometer and the mean cell fluorescence of the stained cells and the percentage of TUNEL-positive cells were reported.

### Cell cycle flow cytometry analysis of MDA-MB-231 Cells

The effect of CP and AP on cell cycle distribution was examined by propidium iodide staining followed by FACS Scan Flow cytometry analysis (Partec PAS, Germany). Briefly, MDA-MB-231 Cells at a density of 3.5 × 10^5^ were harvested after treatment with various concentrations of CP and AP for 24 h. Cell culture medium was removed and cells were trypsinized, recollected and washed with PBS. After centrifugation, pellets were resuspended in ice-cold 70% ethanol and incubated at 4 °C for 24 h. In order to remove interfering RNA molecules, 20 μg/ml RNAase (Sigma-Aldrich) was added to the ethanol-fixed cells in PBS along with 20 μg/ml propidium iodide (Sigma-Aldrich) to stain the DNA content of the cells, and incubated at 37 °C for 30 min in the dark. Finally, the cell cycle distribution in the prepared cells was estimated by measuring cell’s DNA content according to the standard procedure using flow cytometer. FlowJo software (Version 7.6.1.) was used to calculate the Percentage of the cells in the G0/G1, S, or G2/M phases of the cell cycle.

### Measurement of nitric oxide release

Nitric oxide (NO) production in the cells was quantified by determining the accumulation of nitrite in the supernatant of cultured cells by using the Griess reagent (Naphthylethilenediamin (NED) 0.1% and sulfanilamide 2% in phosphoric acid). MDA-MB-231 cells with the density about 1.4 × 10^4^ cells/well were seeded in a 96-well plate, and incubated with 2x IC50 concentrations of CP, and AP. After 12 h, 100 μl of the cell supernatant was picked up, and an equal volume of Vanadium chloride (54 mM in HCl 1 M) and 50 μl Griess reagent was added, and incubated at 37 °C in the dark for 40 min. The optical density of the samples was measured at 540 nm with an ELISA reader (Model wave xs2, BioTek, USA), and the quantity of NO was determined by using standard curve method.

### Intracellular ROS Measurement

CP and AP intracellular ROS generation was measured by DCFH2-DA (2′,7′- dichlorodihydrofluorescin diacetate) (Sigma-Aldrich), a non-fluorescent membrane permeable probe, which oxidizes in the presence of peroxides to the highly fluorescent DCF (2′,7′-dichlorofluorescein), and traps inside the cell. Briefly, MDA-MB-231 cells (1 × 10^5^ cells per well) were treated with CP (400, 800 μg/ml) and AP (100 and 200 μg/ml) for 12 h. 30 minutes prior to the fluorescent intensity measurement by flow cytometry, cells were collected, centrifuged, washed with PBS, subjected to DCFH2-DA (20 μM) and kept in the dark. The amount of ROS was equivalent to DCF production with an excitation at 485–495 nm and emission at 525–530 nm.

### Mitochondrial transmembrane potential evaluation (ΔΨm)

ΔΨm variation in cancer cells were monitored by mitochondria specific Rhodamine123 staining. MDA-MB-231 cells treated with 400, 800 μg/ml CP and 100, 200 μg/ml AP for 12 h were incubated with 400 μl Rhodamine123 (50 µM) for 30 min in the dark and the fluorescent intensity of the Rhodamine123 was detected in flow cytometry FL-1 channel with excitation and emission at 488 and 525–530 nm, respectively.

### RNA isolation, cDNA Synthesis and Real time PCR

MDA-MB-231 cells were cultured in 6 cm plates, and after reaching 80% confluency were treated with 800 and 200 μg/ml of CP, and AP for 4 h, respectively. Total RNA was isolated by TRIzol Reagent according to the manufacturer’s protocol (LifeTechnologies Inc.), and cleaned up with RNase-free DNase I (Bio Basic). Isolated RNA was then quantified spectrophotometrically, and its integrity was verified by agarose gel electrophoresis. Approximately 1 µg of RNA was subjected to Prime Script RT reagent Kit (Takara) and converted into cDNA for real time PCR assay. PCR reaction was performed in a total volume of 20 µl including 50 ng of cDNA, 1x HOT FIREPol EvaGreen qPCR Mix Plus (Solis BioDyne), and 10 µM of each primer. Thermal cycling was performed on a Rotor-Gene Q 6plex Platform (Qiagen) with the following settings: 15 min at 95 °C for initial denaturation, followed by 35 cycles of 15 s at 95 °C, 20 s at 60 °C and 30 s at 72 °C. All reactions were performed in duplicates for each experimental condition, and results were analyzed with Rotor-Gene Q 2.1.0 software (Qiagen). Gal-3 mRNA expression was normalized by PGK (phosphoglycerate kinase) mRNA expression levels, and expressed as fold changes relative to controls using ∆∆Ct method. Primers for galectin-3 and PGK were designed with the assistance of NCBI primer designing tool, Primer-Blast. The nucleotide sequences for the primer pairs were as follows: Galectin-3: Forward 5′GTGAAGCCCAATGCAAACAGA3′, Reverse 5′AGCGTGGGTTAAAGTGGAAGG3′, PGK: Forward 5′GGCATACCTGCTGGCTGGATG3′, Reverse 5′ACAGGACCATTCCACACAATCTGC3′.

### DNA oxidation

In nucleic acids, 8-oxo-2′-deoxyguanosine (8-oxo-dG) is the major lesion caused by oxidation, and a good indicator for measuring DNA oxidation. Genomic DNA was extracted from MDA-MB-231 cells after 2 h treatment with CP (400, 800 μg/ml) and AP (100, 200 μg/ml) as well as untreated controls. Standard phenol/chloroform extraction procedure with a minor modification was used to extract genomic DNA. In order to prevent accidental DNA oxidation during extraction procedure, cell lysis buffer was supplemented with 1 mM ascorbic acid and 1 mM deferoxamine (iron chelator). 5 µg of extracted DNA was then digested with nuclease P1 (sigma, N8630) to release nucleotides, and then dephosphorylated by treating with alkaline phosphatase (NEB, M02903). Prepared samples were subsequently subjected to 8-Oxo detection ELISA kit (Cayman chemicals, 589320) in a total volume of 50 µl according to manufacturer’s protocol. All samples were quantified in duplicates, and 8-oxo-dG concentrations were reported in pg/ml.

### Comet assay

Comet assay under alkaline condition was employed to evaluate single and double strand breaks at single cell level. In brief, 3.5 × 10^5^ MDA-MB-231 cells/well was treated with 2x IC50 concentrations of CP and AP for 2 h. After collection and counting the cells, about 10^4^ cells in a volume of 100 µl were gently pipetted in a 37 °C PBS solution containing 1% LMPA (low melting point agarose), and were spread over a microscope slides precoated with 1% conventional agarose under a coverslip, and chilled in 4 °C for 15 min to solidify. After removal of the coverslip, the slides were submerged for 1 h in cold lysis buffer (10 mM Tris, 2.5 M NaCl, 100 mM EDTA and 1% freshly added Triton X100, pH 10), and then immersed in denaturing buffer (1 mM EDTA and 300 mM NaOH, pH 13) at 4 °C for 30 min to unwind the DNA in the lysed cells. Subsequently, slides were electrophoresed under the same denaturing condition for 30 min at 0.6 V/cm. after electrophoresis, slides were dipped in neutralization solution (0.4 M Tris, pH 7.5) for 5 min, stained with 1x SYBRE green, and imaged by a fluorescent microscope (Axioscope 2 plus, Zeiss, Germany). Open Comet software was used to analyze the Comet images, and the presented data are the results of analysis of at least 100 comets in each sample.

### Cell spheroid Generation from MDA-MB-231 breast cancer cell line

In order to generate suitable cell spheroids for screening the compounds, a combination of hanging drop (HD) and liquid overlay (LO) techniques was used as described previously^61^. In brief, 50 µl drops of a cell suspension with the concentration of about 1.0 × 10^5^ cells/ml (5000 cells/drop) were deposited on the interior surface of a petri dish lid, and were incubated until the spheroids were formed. The spheroids were then transferred to a round bottom 96-well microplate precoated with 50 μl of 0.5% poly-HEMA (sigma) in 95% ethanol, to prevent the cells to adhere to the bottom of the microplate, and were supplemented with 200 µl culture medium to grow for 2 more days.

### Evaluation of cell viability, apoptosis, and cell cycle distribution of the CP treated MDA-MB-231 cell spheroids

To evaluate the cellular viability, cell spheroids were treated for 48 h with 250–2000 μg/ml of CP, and the MTT assay was employed the same as for the 2D cell culture. For estimation of apoptosis, MDA-MB-231 spheroids, pre-selected for volume and shape, were exposed to IC50 concentration of CP (1200 µg/ml) for 24 h, stained with EB/AO, and observed under fluorescent microscope. Furthermore, in order to quantify the apoptotic cells population, CP treated spheroids were trypsinized, and the singled cell suspensions were stained with AnnexinV-FITC/PI. The percentage of apoptotic cells was determined by flow cytometry analysis. The same CP treatment and cells preparation was also used for the cycle analysis, and the test was performed the same as for the 2D cell culture. The cell cycle distribution was analyzed by flowJo software.

### UV Absorption Spectra Measurements

The absorption spectra were acquired on a Cary 100 Bio-model, Australia spectrophotometer. Rat hepatocyte genomic DNA was isolated by standard phenol-chloroform procedure. Isolated DNA was quantified by measuring the absorbance at 260 nm, and its quality was evaluated by both monitoring the A260/A280 ratio ≥1.8, and agarose gel electrophoresis. DNA stock solutions were prepared in 0.1 M phosphate buffer (pH 7.4), and stored at −20 °C. CP, and AP UV Absorption Spectra studies were performed with a fixed concentration of rat hepatocyte DNA (50 μg/ml) in phosphate buffer at room temperature by spectrophotometry over a wavelength range of 190–400 nm. Initially, the absorbance of CP and AP was measured in phosphate buffer at concentrations ranging from 9 to 150 μg/ml, and 9 to 300 μg/ml respectively, the solvent was taken as reference, and then similar procedure was carried out after addition of constant amounts of DNA (50 μg/ml).

### Fluorescence Spectra Measurements

The fluorescence spectra were obtained using a Carry eclipse (Australia) spectrophotometer. Ethidium bromide (EB) fluorescence quenching assay (competitive binding studies) was performed by using DNA-EB system, in which, the fluorescence of the DNA is greatly enhanced by the intercalation of EB between DNA base pairs. The fluorescence quenching was recorded by the addition of different concentrations of CP (0–7.3 μM) and AP (0–8 μM) to a solution containing both 2.6 μM EB and 50 μg/ml DNA. Measurements were done in the range of 530–700 nm upon excitation at 500 nm by applying a 1 cm path length fluorescence cuvette at 310 K. The slit width was set as 10 nm/10 nm for excitation/emission, respectively.

### Circular Dichroism (CD) studies

CD spectra were recorded on a Circular Dichroism 215, Aviv, USA spectrophotometer using a Quartz cuvette with a path length of 1 cm in far-UV range at wavelengths between 220 and 320 nm at room temperature, and pH = 7.4. All the reported CD data were obtained by using 75 μg/ml CP and AP in a 100 μg/ml DNA solution. The changes in CD spectra were monitored against a blank and the spectrum of the buffer solution was recorded and subtracted from the spectra of DNA and DNA-CP/AP complex.

### Statistical Analysis

Experimental data were processed by Microsoft Excel 2013 software. Results were obtained from three or more independent experiments, and presented as mean ± standard deviation. T-test was used to determine the significance between the means, and p-values not greater than 0.05 were considered significant.
